# Commentary: Insights into the Development of the Brief Observation of Social Communication Change (BOSCC)

**DOI:** 10.29245/2578-2959/2018/5.1166

**Published:** 2018-10-09

**Authors:** Rebecca Grzadzinski, Catherine Lord

**Affiliations:** 1 University of North Carolina at Chapel Hill (UNC), Chapel Hill, NC, USA; 2 Carolina Institute for Developmental Disabilities, Carrboro, NC, USA; 3 University of California at Los Angeles (UCLA), Los Angeles, CA, USA

A recent review of early behavioral interventions for autism spectrum disorder^6^ suggested that many intervention trials are of “low quality”^18^, in part due to the limited ability of researchers to interpret and evaluate efficacy. Most early interventions focus on teaching social communication skills, core deficits in children with ASD. However, measures currently used to quantify social communication skills, such as the Autism Diagnostic Observation Schedule (ADOS-2)^14^ were developed to be primarily diagnostic and lack the sensitivity to capture subtle changes over relatively brief periods^2,4^. The field of ASD intervention research needs measures that can identify subtle changes in core ASD symptoms.

Our initial work^9^ provided the first evidence for the reliability and validity of a novel measure known as the Brief Observation of Social Communication Change (BOSCC) to quantify subtle changes in social communication skills. Since the publication of this work, several additional studies have utilized the BOSCC^3,5,11,13,16,17^. The BOSCC is a 15-item measure that was initially developed by expanding and adding to ADOS-2 items. The BOSCC is coded from a videotaped observation of a child during a naturalistic social and play interaction. Our initial work^9^ used a sample of 56 children with minimal language between the ages of 1 and 5 years who were participating in intervention trials. The primary objectives of the study were to assess the inter-rater and test-retest reliability of the BOSCC and the ability of the BOSCC to quantify subtle changes in ASD symptoms. Results showed high inter-rater (Intraclass Correlation Coefficients; ICCs = 0.88–0.98) and test-retest (ICCs = 0.79–0.90) reliabilities as well as statistically significant decreases (improvements) in BOSCC scores, with small to moderate effect sizes (effect sizes = 0.37–0.60), over six to eight months of intervention^9,11^. In contrast, the ADOS Calibrated Severity Score did not change significantly over the same time period. Children who demonstrated improvements in standard scores on the Vineland Adaptive Behavior Scales Communication domain^19^ and Mullen Scales of Early Learning Receptive Language domain^15^ also showed statistically significant decreases on the BOSCC, suggesting that the changes seen in the BOSCC align with changes seen in other commonly used standardized measures.

## Benefits of the BOSCC

The BOSCC is a measure of global ASD change and may be particularly useful in intervention trials that focus on improvements across a range of ASD symptoms. The BOSCC was developed to be flexible for use across different sites, intervention types, and naturalistic social contexts. While our initial work applied the BOSCC coding scheme to parent-child free play videos, a standard administration, conducted by a researcher who is unaware of the child’s treatment status and timepoint, is recommended. This standard administration was developed in order to eliminate potential bias by parents who are aware of the child’s treatment status or timepoint and to provide a context in which changes observed in the child could be distinguished from changes observed in the parent (who may have received training) or the parent-child dyad. We have also had success in applying the BOSCC to other naturalistic social interactions such as segments of ADOS videos^11^. Researchers who are interested in using the BOSCC should evaluate which context would be most feasible and effective for their study, so long as the context is play-based and social.

Video-based coding offers many benefits. Videos capture the totality, richness, and complexity of a behavior, allowing the observer to dissect nuanced behaviors and to explore the dyadic unfolding of social behaviors. Videos also allow for repeated and varied analyses. For example, videos that are used for BOSCC coding may be used for a variety of other purposes, such as coding parent fidelity of intervention or coding other child behaviors unrelated to the BOSCC, such as a child’s motor skills, creating a standardized context for a language sample or focusing on more specific features, such as the use of various consonant sounds. Researchers that have gathered videos from previously conducted intervention trials may also be interested in applying the BOSCC coding scheme to applicable videos retrospectively. Use of video-based observations also provides opportunities for advancements in telemedicine. For example, rural study participants could gather video data in their homes without requiring visits from research staff or attendance at clinics.

Unlike other commonly used measures, such as the ADOS-2, which can require up to 60 minutes of both the participant’s time as well as the time of an experienced clinician, the BOSCC coding scheme is applied to 10- to 12-minute videos, significantly decreasing the burden on participants and highly skilled clinician researchers. Unlike the ADOS-2 and other standardized measures which require a high level of expertise and training to be able to administer and score reliably, the BOSCC was developed to be used reliably by less experienced individuals. The BOSCC can be administered and coded by post-baccalaureate research assistants. On average, with guidance, training, and participation in BOSCC consensus meetings, our trainees typically became reliable within 12 BOSCC videos.

The BOSCC also offers an appropriate context in which to employ automated algorithms or body sensors that record physiological activity (e.g., skin conductance, heart rate) to detect change. For example, we are working with researchers with expertise in machine learning techniques to generate automated coding of change over time using audio recordings from BOSCC sessions. This work would provide new approaches to capturing change that would not rely on human observation alone, but rather combine multiple methods (human observation and computer automated processes) to maximally identify changes in response to intervention.

## Limitations of the BOSCC

Results of the initial study were promising but limitations highlight the preliminary nature of this work. Our first study was based on a relatively small sample of children, although many other researchers have now taken an interest in using the BOSCC and are exploring its utility in new, larger samples. While the BOSCC was developed to be flexibly applied to a range of playful social contexts, researchers should be mindful of the limitations of certain contexts and interacting partners. For example, when applying the BOSCC coding scheme to parentchild interactions for parent mediated interventions, it is important to keep in mind that changes in BOSCC scores could be related to changes in parental behavior or in the parent-child relationship. Similarly, the BOSCC may not be independent of other changes in the child’s development, such as changes in language or cognitive skills. This highlights the importance of ensuring consistency between pre and post observations (e.g., same social context, same environment, same materials) in order to most accurately capture the child’s behavioral change separate from other changes that may be occurring.

Though the short length of BOSCC administrations is a benefit, the BOSCC results are, consequently, a very brief snapshot of the child’s behavior, a limitation also present with the lengthier ADOS-2. Though short observations are commonly used to assess change in early intervention trials^7,8^, the question of whether we can truly gather a representative sample of the child’s behavior within such a short period of time remains unanswered. There are ways to promote a useful snapshot of the child’s behavior within a short time frame. For example, researchers can ensure that the interaction is comfortable, fun and natural by giving the child “warm-up” time with the play partner before the BOSCC administration begins, confirming that the child is not tired or ill when the sample is gathered, and asking caregivers whether the sample gathered was representative of the child’s behavior. There may be instances when a second video sample should be gathered or when a video sample should not be used, such as if the child is unusually irritable.

Our data suggested that researchers should be cautious when using the four BOSCC items in the RRB domain. First, research assistants with less experience with ASD may require more training to be able to reliably identify RRB behaviors compared to Social Communication behaviors. Second, these items showed highly skewed distributions such that the majority of children did not demonstrate these behaviors during the time-limited BOSCC sample. These results are consistent with RRB items on the ADOS-2 over a longer period and with other studies that have highlighted the difficulty with measuring RRBs by observation^1,10,20^. Some researchers have suggested that accurate observation of RRBs may require multiple observations in different contexts or may need to incorporate parent report^12,20^. Though our direct observation method may have been influenced by possible novelty of materials at the first time point^10^, our initial results found stability in RRBs over short periods of time (test-retest reliability was adequate), countering this possibility. Nevertheless, additional research should continue to explore ways to accurately observe and quantify RRBs in children with ASD as well as to maximize the benefits and minimize the limitations of direct observation of RRBs.

## Next Steps

While we hope the BOSCC will help researchers identify efficacious interventions, the BOSCC will not answer *all* questions for *all* interventions. The BOSCC coding scheme may not adequately quantify changes during interventions that are of low intensity, occur over a very short period of time, or whose primary teaching focus is on a highly specific skill (e.g., pointing) or non-ASD-specific skill (e.g., gait)^16^. Though it is likely that some participants will not demonstrate change or will not “respond” to treatment, it is also possible that participants will not generalize improvements made in treatment to the video context in which the BOSCC was applied. For example, if the intervention is conducted in school by teachers, the BOSCC may not show significant changes when applied to parentchild interactions at home.

Our initial work focused on toddlers and preschoolers who were minimally verbal, or equivalent to ADOS-2 Toddler or Module 1. We anticipate that the BOSCC will follow a similar modular format based on age and language level as the ADOS-2. The BOSCC coding scheme that is applicable to verbally-fluent individuals with ASD is currently under development. While the BOSCC was developed to be applicable to children with ASD, it is possible that the coding scheme may be effective at capturing change in children with other neurodevelopmental disorders, particularly those with overlapping social and/or communication difficulties. For example, researchers outside our lab are currently exploring the utility of the BOSCC in a sample with Fragile X. Studies like this will contribute to our understanding of the efficacy of the BOSCC in non-ASD populations.

Our work and the work of other researchers will continue to contribute to our understanding of the benefits and limitations of the BOSCC. Researchers who are interested in using the BOSCC may attend trainings to learn about the administration and coding of the BOSCC. We are also in the process of developing an online coding system, which would allow researchers to upload videos to be coded by BOSCC-reliable research assistants. Researchers who choose to use the online coding system would not need to attend a BOSCC coding training, would not need to train coders in their own lab, and could receive truly “blinded” feedback without going to extensive lengths to keep examiners and coders separate. An online coding system would also improve clinical applicability of the BOSCC by allowing clinicians to submit videos without needing to receive training or score videos themselves in order to have a reliable metric of change.

Questions remain about the utility of the BOSCC in quantifying changes in ASD symptoms. We will continue to explore, as will other researchers, what is a “clinically meaningful” amount of change on the BOSCC and when, over the course of an intervention, we might expect to capture this amount of change. Future research will assess what it means for interventions if the individual does not show a clinically meaningful decrease on the BOSCC as well as when changes in the BOSCC do not align with clinical judgment or changes in other standard measures. The BOSCC is one of many steps in the development of innovative ways to identify beneficial treatments for individuals with ASD.

## References

[1] Barber AB, Wetherby AM, Chambers NW. Brief report: Repetitive behaviors in young children with autism spectrum disorder and developmentally similar peers: A follow up to Watt et al. (2008). Journal of autism and developmental disorders. 2012; 42(9): 2006–2012. 22222776 10.1007/s10803-011-1434-3

[2] Dawson G1, Rogers S, Munson J, Randomized, controlled trial of an intervention for toddlers with autism: the Early Start Denver Model. Pediatrics. 2010; 125(1): e17–e23. doi:10.1542/peds.2009-0958. 19948568 PMC4951085

[3] Divan G, Vajaratkar V, Cardozo P, The Feasibility and Effectiveness of PASS Plus, A Lay Health Worker Delivered Comprehensive Intervention for Autism Spectrum Disorders: Pilot RCT in a Rural Low and Middle Income Country Setting. Autism Research, in press. 2018. 10.1002/aur.1978 30095230

[4] Estes A, Munson J, Rogers SJ, Long-term outcomes of early intervention in 6-year- old children with autism spectrum disorder. Journal of the American Academy of Child and Adolescent Psychiatry. 2015; 54(7): 580–587. 26088663 10.1016/j.jaac.2015.04.005 PMC4475272

[5] Fletcher-Watson S, Petrou A, Scott-Barrett J, A trial of an iPad™ intervention targeting social communication skills in children with autism. Autism. 2016; 20(7): 771–782. 26503990 10.1177/1362361315605624 PMC5015758

[6] French L, Kennedy EM. Annual Research Review: Early intervention for infants and young children with, or at-risk of, autism spectrum disorder: a systematic review. Journal of Child Psychology and psychiatry. 2018; 59(4): 444–456. 29052838 10.1111/jcpp.12828

[7] Goods KS, Ishijima E, Chang YC, Preschool based JASPER intervention in minimally verbal children with autism: Pilot RCT. Journal of autism and developmental disorders. 2013; 43(5): 1050–1056. 22965298 10.1007/s10803-012-1644-3 PMC4222903

[8] Green J, Charman T, McConachie H, Parent-mediated communication-focused treatment in children with autism (PACT): a randomised controlled trial. The Lancet. 2010; 375(9732): 2152–2160. 10.1016/S0140-6736(10)60587-9 PMC2890859 20494434

[9] Grzadzinski R, Carr T, Colombi C, Measuring changes in social communication behaviors: preliminary development of the Brief Observation of Social Communication Change (BOSCC). Journal of autism and developmental disorders. 2016; 46(7): 2464–2479. 27062034 10.1007/s10803-016-2782-9

[10] Harrop C, McConachie H, Emsley R, Restricted and repetitive behaviors in autism spectrum disorders and typical development: Cross-sectional and longitudinal comparisons. Journal of autism and developmental disorders. 2014; 44(5): 1207–1219. 24234675 10.1007/s10803-013-1986-5

[11] Kim S, Grzadzinski R, Martinez K, Measuring treatment response in children with autism spectrum disorder: Applications of the Brief Observation of Social Communication Change to the Autism Diagnostic Observation Schedule. Autism, 1362361318793253. 10.1177/1362361318793253 PMC6522321 30303398

[12] Kim SH, Lord C. Restricted and repetitive behaviors in toddlers and preschoolers with autism spectrum disorders based on the Autism Diagnostic Observation Schedule (ADOS). Autism Research. 2010; 3(4): 162–173. 20589716 10.1002/aur.142 PMC3005305

[13] Kitzerow J, Teufel K, Wilker C, Using the brief observation of social communication change (BOSCC) to measure autism-specific development. Autism Research. 2015. 10.1002/aur.1588 26643669

[14] Lord C, Rutter M, DiLavore P, Autism diagnostic observation schedule– 2nd edition (ADOS-2). Los Angeles, CA: Western Psychological Corporation 2012.

[15] Mullen EM. Mullen scales of early learning. Circle Pines, MN: American Guidance Service 1995.

[16] Nordahl-Hansen A, Fletcher-Watson S, McConachie H, Relations between specific and global outcome measures in a social-communication intervention for children with autism spectrum disorder. Research in Autism Spectrum Disorders. 2016; 29: 19–29.

[17] Pijl MK, Rommelse NN, Hendriks M, Does the Brief Observation of Social Communication Change help moving forward in measuring change in early autism intervention studies. Autism. 2016; 1362361316669235. 10.1177/1362361316669235 29148287

[18] Simonoff E Commentary: Randomized controlled trials in autism spectrum disorder: state of the field and challenges for the future. Journal of Child Psychology and Psychiatry. 2018; 59(4): 457–459. 29574736 10.1111/jcpp.12905

[19] Sparrow SS, Cicchetti DV, Balla DA. Vineland adaptive behavior scales, (Vineland-II). Circle Pines, MN: American Guidance Services 2005.

[20] Watt N, Wetherby AM, Barber A, Repetitive and stereotyped behaviors in children with autism spectrum disorders in the second year of life. Journal of autism and developmental disorders. 2008; 38(8): 1518–1533. 18266099 10.1007/s10803-007-0532-8 PMC2637365

